# Comparative mouse lung injury by nickel nanoparticles with differential surface modification

**DOI:** 10.1186/s12951-018-0436-0

**Published:** 2019-01-07

**Authors:** Yiqun Mo, Mizu Jiang, Yue Zhang, Rong Wan, Jing Li, Chuan-Jian Zhong, Huangyuan Li, Shichuan Tang, Qunwei Zhang

**Affiliations:** 1 Department of Environmental and Occupational Health Sciences, School of Public Health and Information Sciences, University of Louisville, 485 E. Gray Street, Louisville, KY 40209 USA; 2 Department of Gastroenterology, Children’s Hospital, Zhejiang University School of Medicine, Hangzhou, P. R. of China; 3 Department of Pathology, Fujian Medical University, Fuzhou, P. R. of China; 4 Department of Chemistry, State University of New York at Binghamton, Binghamton, NY 13902 USA; 5 Department of Preventive Medicine, Fujian Provincial Key Laboratory of Environment Factors and Cancer, School of Public Health, Fujian Medical University, Fuzhou, P. R. of China; 6 Beijing Municipal Institute of Labor Protection, Beijing, P. R. of China

**Keywords:** Nickel nanoparticles (Nano-Ni), Partially passivated Nano-Ni (Nano-Ni–P), Carbon-coated Nano-Ni (Nano-Ni–C), Bronchoalveolar lavage (BAL), Bronchoalveolar lavage fluid (BALF), Thiobarbituric acid reactive substances (TBARS), 8-Hydroxy-2′-deoxyguanosine (8-OHdG), Matrix metalloproteinase-2 and -9 (MMP-2/9)

## Abstract

**Background:**

Previous studies have demonstrated that exposure to nickel nanoparticles (Nano-Ni) causes oxidative stress and severe, persistent lung inflammation, which are strongly associated with pulmonary toxicity. However, few studies have investigated whether surface modification of Nano-Ni could alter Nano-Ni-induced lung injury, inflammation, and fibrosis in vivo. Here, we propose that alteration of physicochemical properties of Nano-Ni through modification of Nano-Ni surface may change Nano-Ni-induced lung injury, inflammation, and fibrosis.

**Methods:**

At first, dose–response and time-response studies were performed to observe lung inflammation and injury caused by Nano-Ni. In the dose–response studies, mice were intratracheally instilled with 0, 10, 20, 50, and 100 μg per mouse of Nano-Ni and sacrificed at day 3 post-exposure. In the time-response studies, mice were intratracheally instilled with 50 µg per mouse of Nano-Ni and sacrificed at days 1, 3, 7, 14, 28, and 42 post-instillation. At the end of the experiment, mice were bronchoalveolar lavaged (BAL) and the neutrophil count, CXCL1/KC level, LDH activity, and concentration of total protein in the BAL fluid (BALF) were determined. In the comparative studies, mice were intratracheally instilled with 50 μg per mouse of Nano-Ni or with the same molar concentration of Ni as Nano-Ni of either partially [O]-passivated Nano-Ni (Nano-Ni–P) or carbon-coated Nano-Ni (Nano-Ni–C). At day 3 post-exposure, BAL was performed and the above cellular and biochemical parameters in the BALF were analyzed. The MMP-2/9 protein levels and activities in the BALF and mouse lung tissues were also determined. Mouse lung tissues were also collected for H&E staining, and measurement of thiobarbituric acid reactive substances (TBARS) and 8-hydroxy-2′-deoxyguanosine (8-OHdG) in the genomic DNA. At day 42 post-exposure, mouse right lung tissues were collected for H&E and Trichrome stainings, and left lung tissues were collected to determine the hydroxyproline content.

**Results:**

Exposure of mice to Nano-Ni resulted in a dose–response increase in acute lung inflammation and injury reflected by increased neutrophil count, CXCL1/KC level, LDH activity, and concentration of total protein in the BALF. The time-response study showed that Nano-Ni-induced acute lung inflammation and injury appeared as early as day 1, peaked at day 3, and attenuated at day 7 post-instillation. Although the neutrophil count, CXCL1/KC level, LDH activity, and concentration of total protein in the BALF dramatically decreased over the time, their levels were still higher than those of the controls even at day 42 post-exposure. Based on the results of the dose- and time-response studies, we chose a dose of 50 µg per mouse of Nano-Ni, and day 3 post-exposure as short-term and day 42 post-exposure as long-term to compare the effects of Nano-Ni, Nano-Ni–P, and Nano-Ni–C on mouse lungs. At day 3 post-exposure, 50 μg per mouse of Nano-Ni caused acute lung inflammation and injury that were reflected by increased neutrophil count, CXCL1/KC level, LDH activity, concentration of total protein, and MMP-2/9 protein levels and activities in the BALF. Nano-Ni exposure also caused increased MMP-2/9 activities in the mouse lung tissues. Histologically, infiltration of large numbers of neutrophils and macrophages in the alveolar space and interstitial tissues was observed in mouse lungs exposed to Nano-Ni. Nano-Ni–P exposure caused similar acute lung inflammation and injury as Nano-Ni. However, exposure to Nano-Ni–C only caused mild acute lung inflammation and injury. At day 42 post-exposure, Nano-Ni caused extensive interstitial fibrosis and proliferation of interstitial cells with inflammatory cells infiltrating the alveolar septa and alveolar space. Lung fibrosis was also observed in Nano-Ni–P-exposed lungs, but to a much lesser degree. Only slight or no lung fibrosis was observed in Nano-Ni–C-exposed lungs. Nano-Ni and Nano-Ni–P, but not Nano-Ni–C, caused significantly elevated levels of TBARS in mouse lung tissues and 8-OHdG in mouse lung tissue genomic DNA, suggesting that Nano-Ni and Nano-Ni–P induce lipid peroxidation and oxidative DNA damage in mouse lung tissues, while Nano-Ni–C does not.

**Conclusion:**

Our results demonstrate that short-term Nano-Ni exposure causes acute lung inflammation and injury, while long-term Nano-Ni exposure causes chronic lung inflammation and fibrosis. Surface modification of Nano-Ni alleviates Nano-Ni-induced pulmonary effects; partially passivated Nano-Ni causes similar effects as Nano-Ni, but the chronic inflammation and fibrosis were at a much lesser degree. Carbon coating significantly alleviates Nano-Ni-induced acute and chronic lung inflammation and injury.

## Background

The application of nanomaterials is becoming increasingly extensive following the rapid development of nanotechnology. Concurrently, the nanotechnology workforce is rapidly growing, with an anticipated 2 million in nanotechnology in the US by 2020, with 6 million total workers worldwide [1]. Metal nanoparticles are widely used in industrial applications, ceramics, cosmetics, and biological medicine due to their specific optical, mechanical, and electrical properties that differ from those of bulk materials. Consequently, public concerns about the biological effects of nanomaterials on the environment and on human health are warranted.

Nickel is a d^8^ transition metal; nickel nanoparticles (Nano-Ni) have specific characteristics, such as high reactivity, high magnetism, high surface area, low melting point, and low autoignition temperature [2, 3]. They are widely used in industry as printing inks, ceramics, and catalysts, and in the electrical and electronics industry for their magnetic and optical properties [4–6]. Nickel nanoparticles are suitable for environmental applications such as adsorption of hazardous dyes and inorganic pollutants and thus can play a role in decontamination [7, 8]. Nickel nanoparticles show cytotoxicity against cancerous cells, evident from the distortion of their morphology [9–11]. Nickel-alloy nanomaterials have received special interest in biomedical applications. Magnetic metal nanoparticles, typically composed of iron, cobalt, and nickel, have been adopted for MRI applications [12–14]. The increased use of Nano-Ni has led to increased environmental and occupational exposure, which is associated with an elevated risk for skin allergies, lung inflammation, lung fibrosis, pneumonitis, emphysema, alveolar proteinosis, and cancer of the respiratory tract [15, 16]. A case report involving exposure to Nano-Ni by spraying Nano-Ni onto bushes for turbine bearings using a metal arc process resulted in the worker dying of adult respiratory distress syndrome (ARDS) at day 13 post-exposure [17]. Another case report involved a worker developing nickel sensitization while working with nickel nanoparticles without special respiratory protection or control measures [18]. The potential health effects of nickel nanoparticles cannot be ignored; it is essential to study their toxic effects and explore potential ways to reduce them. Modification of physicochemical properties such as surface area of Nano-Ni may potentially reduce its toxicity.

The pulmonary toxicity of nickel particles in both occupational and non-occupational settings is not only proportional to the concentration of metal exposure, but also to their particle sizes. Previous studies have shown that the toxicity of metal nanoparticles is associated with many factors, such as density, shape, chemical composition, phase, and surface properties [19–24]. Metal nanoparticles have wide range of physical and chemical properties that can alter their in vivo activity [24, 25]. Scale, surface area to mass ratio, and surface charge can all affect the interactions of nanoparticles with a host organism. Our previous studies have demonstrated that Nano-Ni with mean diameter of 20 nm instilled into the rat lungs caused a greater inflammatory response as compared to that of standard-sized nickel particles [3]. The small diameter and subsequent high particle numbers of Nano-Ni appear to play an important role in Nano-Ni-induced toxicity [2, 26].

Our previous studies have shown that exposure of rats to a low dose of Nano-Ni leads to severe and persistent lung inflammation and injury even at 30 days post-exposure [3]. In addition, our and other in vitro and in vivo studies have shown that exposure to nickel or nickel-containing nanoparticles causes oxidative stress, inflammation, apoptosis, nuclear accumulation of hypoxia inducible factor 1 α (HIF-1α), activated matrix metalloproteinases (MMPs), and DNA damage [2, 3, 27–29]. Previous studies have suggested that the pro-inflammatory response and cytokine production are involved in Nano-Ni-induced lung inflammation, injury, and fibrosis [3, 30–32].

The aim of this study was to examine whether surface modification of Nano-Ni could alter Nano-Ni-induced lung inflammation and injury in vivo. Here, we chose the same size of Nano-Ni, Nano-Ni partially passivated with [O] (Nano-Ni–P), and Nano-Ni coated with carbon (Nano-Ni–C) to compare their ability to cause lung inflammation, injury, and fibrosis. We determined whether surface modification of Nano-Ni such as carbon-coating could reduce Nano-Ni-induced pulmonary toxicity.

## Materials and methods

### Nickel nanoparticles and their characterization

**Fig. 1:**
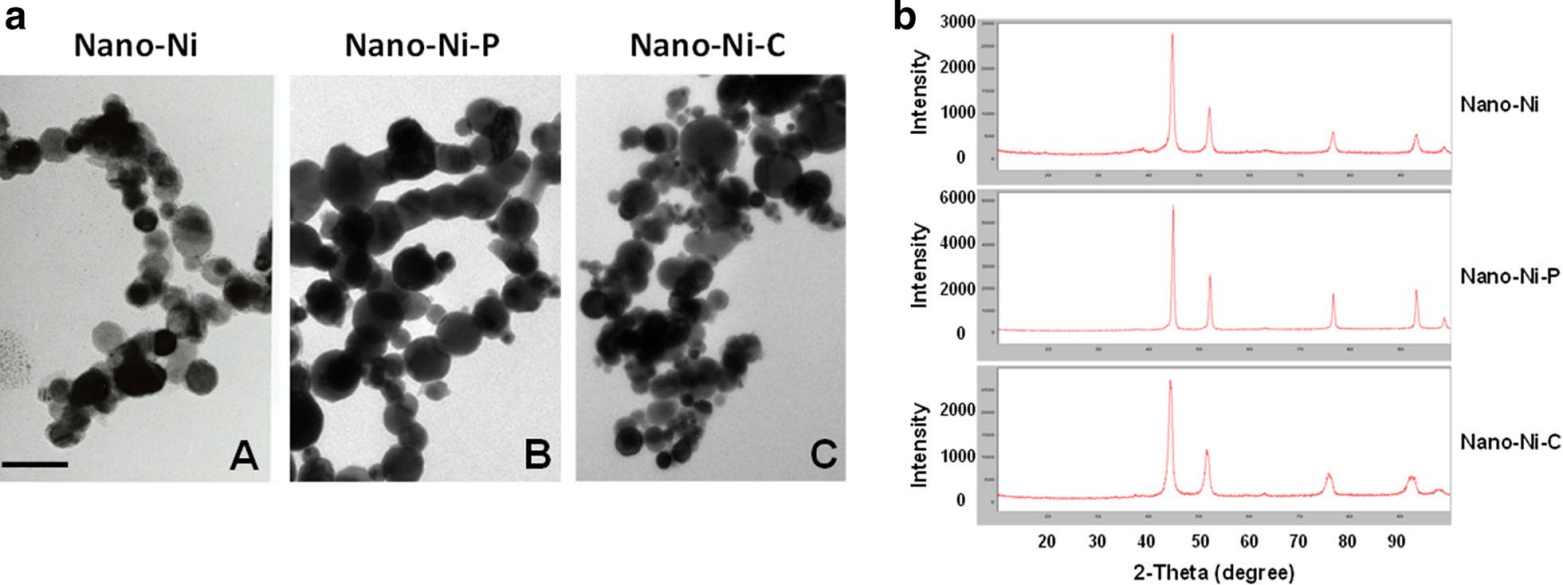
A representative set of TEM images (**a**) and XRD patterns (**b**) for nickel nanoparticles. **a** Images were captured by transmission electron microscopy (TEM). Bar in A represents 100 nm for all panels. **b** XRD patterns show the differences in the peak positions and peak widths

Nano-Ni with a mean diameter of 20 nm was provided by Inabata & Co., Ltd., Vacuum Metallurgical Co., Ltd., Japan. Both Nano-Ni–P and Nano-Ni–C with a mean diameter of 20 nm were purchased from US Research Nanomaterials, Inc., Houston, TX, USA, made by laser evaporation. Nano-Ni–P was partially passivated with [O] (0.85 wt%), while Nano-Ni–C was coated with a carbon layer of 0.47 nm in thickness (C = 3.61 wt%, O < 0.3%). The microstructure, composition, and characteristics of Nano-Ni, Nano-Ni–P, and Nano-Ni–C were determined by transmission electron microscopy (TEM) (Fig. 1a) and X-ray powder diffraction (XRD) (Fig. 1b). TEM measurements were performed on a JEOL JEM-2100F (Tokyo, Japan), operated at an accelerating voltage of 200 kV. The average hydrodynamic sizes of nickel nanoparticles were measured by dynamic light scattering (DLS) in a Zetasizer Nano ZS (model ZEN3600), equipped with a 4.0 mV 633 nm laser (Malvern Instruments, Malvern, UK). The characterization of these nickel nanoparticles was summarized in Table 1. Briefly, the specific surface area is 43.8 m^2^/g for Nano-Ni, and 40.0 ~ 60.0 m^2^/g for both Nano-Ni–P and Nano-Ni–C. Hydrodynamic diameter measured by DLS is 250 nm for Nano-Ni, 246.4 nm for Nano-Ni–P, and 274.8 nm for Nano-Ni–C. The Zeta potential is 2.0 ± 1.4 mV for Nano-Ni, 2.3 ± 1.6 mV for Nano-Ni–P, and 2.2 ± 1.6 mV for Nano-Ni–C. The solubility of Nano-Ni (24.01 ± 0.95 ppm), Nano-Ni–P (5.66 ± 0.18 ppm), and Nano-Ni–C (12.97 ± 0.15 ppm) in physiological saline was determined by inductively coupled plasma-atomic emission spectrometry (ICP-AES) as previously reported [29, 33, 34]. Nano-Ni, Nano-Ni–P, and Nano-Ni–C were dispersed in physiological saline, and ultrasonicated for 10 min and vibrated thoroughly prior to each experiment.

**Table 1:** Characterization of nickel nanoparticles

Metal	Particle size in powder (diameter) (nm, average)	TEM diameter (nm)	DLS diameter (nm)	Specific surface area (m^2^/g)	Zeta potential in H_2_O (mV ± SD)
Nano-Ni	20	10–30	250.0	43.8	2.0 ± 1.4
Nano-Ni–P	20	10–30	246.4	40.0–60.0	2.3 ± 1.6
Nano-Ni–C	20	10–30	274.8	40.0–60.0	2.2 ± 1.6

### Animals

Eight-week-old male C57BL/6 mice, weighing about 22–28 g, were obtained from The Jackson Laboratory (Bar Harbor, ME, USA). The mice were housed in an air-conditioned room (temperature of 20 ± 2 °C, relative humidity of 60 ± 10%) with a 12-h light and 12-h dark cycle environment and with free access to food and water. The mice were allowed to acclimatize for 1–2 weeks before starting the experimental protocol and were monitored daily for general health. Animal use was reviewed and approved by the University of Louisville Institutional Animal Care and Use Committee.

### Exposure of mice to nickel nanoparticles

Mice were exposed to nickel nanoparticles by intratracheal instillation as described previously [2, 3, 34]. The intratracheal instillation model was selected instead of an inhalation study because the former is an easy and reliable method to identify nanoparticle toxicity and to compare responses to different particle types [35]. Mice were grouped randomly. Mouse neck skin was opened by a small midline incision and the trachea was isolated. In the dose–response studies, mice were instilled intratracheally with 10, 20, 50, and 100 µg per mouse of Nano-Ni in 50 µL of physiological saline by a syringe with a 28G1/2 needle under anesthesia, followed by 100 µL of air to ensure deposition of the particles in the lower airways [34]. In the time-response studies, each mouse was instilled intratracheally with 50 µg of Nano-Ni. In the comparative studies, each mouse was instilled intratracheally with 50 µg of Nano-Ni, or with the same molar concentration of Ni as Nano-Ni of either Nano-Ni–P or Nano-Ni–C. Control mice were injected with 50 µL of physiological saline. Immediately after instillation, the skin was closed with a monofilament nylon suture. After surgery, the mice were placed on a warming plate and returned to their cages when they were sternal and ambulatory. Sutures were removed in 7–10 days. The mice were sacrificed at different times after instillation of nickel nanoparticles.

### Bronchoalveolar lavage (BAL) and preparation of bronchoalveolar lavage fluid (BALF)

At the end of the experiment, mice were anesthetized by an overdose injection of tribromoethanol into the abdominal cavity and the abdominal aorta was severed. The trachea was clearly visualized after dissection of skin and soft tissues, and a 20G cannula was inserted into the trachea. 0.5 mL of ice-cold 1× PBS containing 0.4 mM EDTA was used to lavage the lungs bilaterally. The lavage fluid was retrieved by gentle massage. The procedure was repeated another five times. The first and the second collected lavage fluid samples were pooled together and centrifuged at 200*g* for 5 min at 4 °C. The supernatant was collected and stored at − 80 °C for subsequent analysis of biochemical markers and MMP-2/9 protein levels and activities. The cells in the BALF were pooled together. The number of total cells in BALF was calculated based on the number of cells counted under microscope using a hemacytometer. The cell differentiation was evaluated on a cytospin slide stained with Giemsa and May–Grünwald stains (Sigma-Aldrich, St. Louis, MO, USA).

### Evaluation of LDH, total protein, CXCL1/KC, MMP-2, and MMP-9 levels in BALF

The levels of lactate dehydrogenase (LDH) activity, total protein, chemokine (C-X-C motif) ligand 1 (CXCL1)/keratinocyte chemoattractant (KC), and matrix metalloproteinase-2 and -9 (MMP-2 and MMP-9) in BALF were evaluated by commercially available kits according to the manufacturer’s instructions. LDH activity in the BALF was measured by a LDH Cytotoxicity Detection Kit (TaKaRa Bio Inc., Shiga, Japan). The concentration of total protein in the BALF was determined by the Bradford method using Bio-Rad Protein Assay (Hercules, CA, USA). The level of CXCL1/KC was assessed by a Mouse CXCL1/KC PicoKine™ ELISA Kit (Boster Biological, Pleasanton, CA, USA). The MMP-2 and MMP-9 protein levels in BALF were determined by Mouse MMP-2 or MMP-9 PicoKine™ ELISA Kit (Boster Biological, Pleasanton, CA, USA).

### Gelatin zymography assay

MMP-2 and MMP-9 activities in the BALF and lung tissues were determined by a gelatin zymography assay as described previously [29, 36, 37]. The BALF was collected as described above and 48 µL of BALF was loaded in each lane of 10% SDS-PAGE copolymerized with 0.5 mg/mL gelatin, which was used as the substrate under non-reducing conditions. Lung tissues were homogenized in ice-cold 1x PBS with 1× Halt™ Protease Inhibitor Cocktail (Thermo Scientific, Rockford, IL, USA) by using a Tissue-Tearor homogenizer (BioSpec Products, Bartlesville, OK, USA). After the homogenate was centrifuged at 10,000*g* for 15 min at 4 °C, the supernatant was collected and the protein concentration was determined by using Bio-Rad Protein Assay (Hercules, CA, USA). 30 µg protein per lane was subjected to electrophoresis on 10% SDS-PAGE copolymerized with 0.5 mg/mL gelatin. After electrophoresis, the gels were washed at room temperature in 50 mM Tris–HCl buffer (pH 7.5) containing 2.5% Triton X-100 (Sigma, St. Louis, MO, USA) for 1 h, changing solution every 15 min. The gels were then incubated at 37 °C overnight in 50 mM Tris–HCl buffer (pH 7.5) containing 0.2 M NaCl, 7.55 mM CaCl_2_, 1 µM ZnCl_2_, and 1% Triton X-100, with gentle shaking, to develop the enzyme activity bands. After washing with distilled water twice, 5 min each time, the gels were stained with 0.1% Coomassie Brilliant Blue R-250 (Bio-Rad, Hercules, CA, USA) and destained with 10% acetic acid until the clear bands were observed against the background of Coomassie Brilliant Blue-stained gelatin.

### Histological examination

At the end of the experiment, mouse lung tissues were collected, fixed with 10% neutral buffered formalin, dehydrated stepwise through an ascending series of alcohol solutions, and finally degreased in xylene. The tissues were then embedded in paraffin, sectioned at 5 μm by a microtome (Thermo Scientific, Rockford, IL, USA), and stained with hematoxylin and eosin (HE) stains. Images were digitally captured and analyzed using ImageJ (http://imagej.nih.gov/ij/). The volume fraction of lung parenchyma with chronic inflammation in the nickel nanoparticle-exposed mice was quantified by morphometric analysis as described previously [38].

### Trichrome staining

In order to observe whether nickel nanoparticle exposure can cause lung fibrosis, Masson’s Trichrome for Connective Tissue kit (Electron Microscopy Sciences, Hatfield, PA) was used to stain the mouse lung tissues according to the manufacturer’s instructions with minor modification. Lung tissues were deparaffinized, hydrated stepwise through a descending series of alcohol solutions and distilled water, and mordanted in Bouin’s fixative at room temperature overnight to intensify the final coloration. After being washed, tissues were stained in Weigert’s Iron Hematoxylin working solution for 4 min and in Biebrich Scarlet-Acid Fuchsin for 4 min, with the washing between two steps. After rinsing with deionized water, tissues were treated with Phosphomolybdic Acid/Phosphotungstic Acid for 15 min, followed by staining in Aniline Blue solution for 15 min. After briefly being differentiated in 1% acetic acid, tissues were dehydrated stepwise through an ascending series of alcohol solutions, cleared in xylene, and mounted.

### Hydroxyproline assay

To compare the total amount of collagen in lung tissues after nickel nanoparticle exposure, hydroxyproline content in mouse left lungs was measured as previously described [39]. This method is based on the acid hydrolysis of the lung tissue and subsequent determination of the free hydroxyproline in hydrolyzate. Briefly, left lung tissues were hydrolyzed in 1 mL of 6 N HCl at 100 °C overnight. After cooling to room temperature, the hydrolysate was neutralized with 6 N NaOH to approximately pH 6.0. Then 3% chloramine-T (Sigma-Aldrich, St. Louis, MO, USA) solution containing 50% isopropanol and 0.5 M sodium acetate (pH 6.0) was added to oxidize the free hydroxyproline for the production of a pyrrole, followed by the addition of perchloric acid (Sigma-Aldrich, St. Louis, MO, USA). The addition of Ehrlich’s reagent (5% of 4-dimethylaminobenzaldehyde in methanol) resulted in the formation of a chromophore that can be measured at 560 nm. The amount of hydroxyproline was determined against a standard curve generated by using known concentrations of hydroxyproline (Sigma-Aldrich, St. Louis, MO, USA).

### Thiobarbituric acid reactive substances (TBARS) assay

TBARS assay was used to determine lipid peroxidation in the lung tissues after nickel nanoparticle exposure as previously reported [40, 41]. Briefly, frozen lung tissues were weighed and homogenized in 20 volumes of ice-cold 1x PBS with 1x Halt™ Protease Inhibitor Cocktail (Thermo Scientific, Rockford, IL, USA) by using a Tissue-Tearor homogenizer (BioSpec Products, Bartlesville, OK, USA). After the homogenate was centrifuged at 1600*g* for 10 min at 4 °C, the supernatant was collected and the protein concentration was determined by using Bio-Rad Protein Assay (Hercules, CA, USA) by using bovine serum albumin (BSA) as a standard protein. To determine lipid peroxidation, 0.25 mL of the supernatant was extracted with trichloroacetic acid (TCA) and reacted with TBA at 100 °C for 15 min after mixing with 0.5 mL of freshly prepared solution containing 0.375% TBA, 15% TCA, and 0.25 N HCl. After cooling, the mixture was centrifuged at 1600*g* for 10 min at room temperature. The absorbance of 200 µL supernatant was measured at 532 nm against a blank. TBARS were calculated by using an extinction coefficient ε of 1.56 × 10^5^ M^−1^ cm^−1^ [42] according to Bear’s Law that Absorbance (OD_532_) equals εLc, where L is the length of light path, and c is the concentration of a substance. The TBARS were expressed as the amount of MDA (nmol) in 1 mg of protein.

### Determination of 8-OHdG level

8-OHdG level was determined in the genomic DNA of mouse lung tissues with or without nickel nanoparticle exposure as described in our previously published report [34]. Genomic DNA was purified by using a QIAamp DNA Mini kit (QIAGEN, Germantown, MD, USA) according to the manufacturer’s instructions, and its concentration was determined by a spectrophotometer (Beckman Coulter, Fullerton, CA, USA). 8-OHdG level in genomic DNA was determined by Oxi-Select™ Oxidative DNA Damage ELISA kit (Cell Biolabs, San Diego, CA, USA) according to the manufacturer’s instruction, and expressed as the amount of 8-OHdG (pg) in 1 μg of genomic DNA.

### Statistical analysis

Data were expressed as the mean ± SE. Differences among groups were evaluated by one-way analysis of variance (ANOVA) followed by Bonferroni t-test, Dunnett’s t-test, or Dunn’s method. If necessary, transformation of data (log or square root) was used to achieve normally distributed data before ANOVA analysis. If a p value was less than 0.05, a difference was considered statistically significant. Statistical analyses were carried out by using SigmaPlot 13.0 software (Systat Software, Inc., San Jose, CA, USA).

## Results

### Dose- and time- response studies with Nano-Ni exposure

**Fig. 2:**
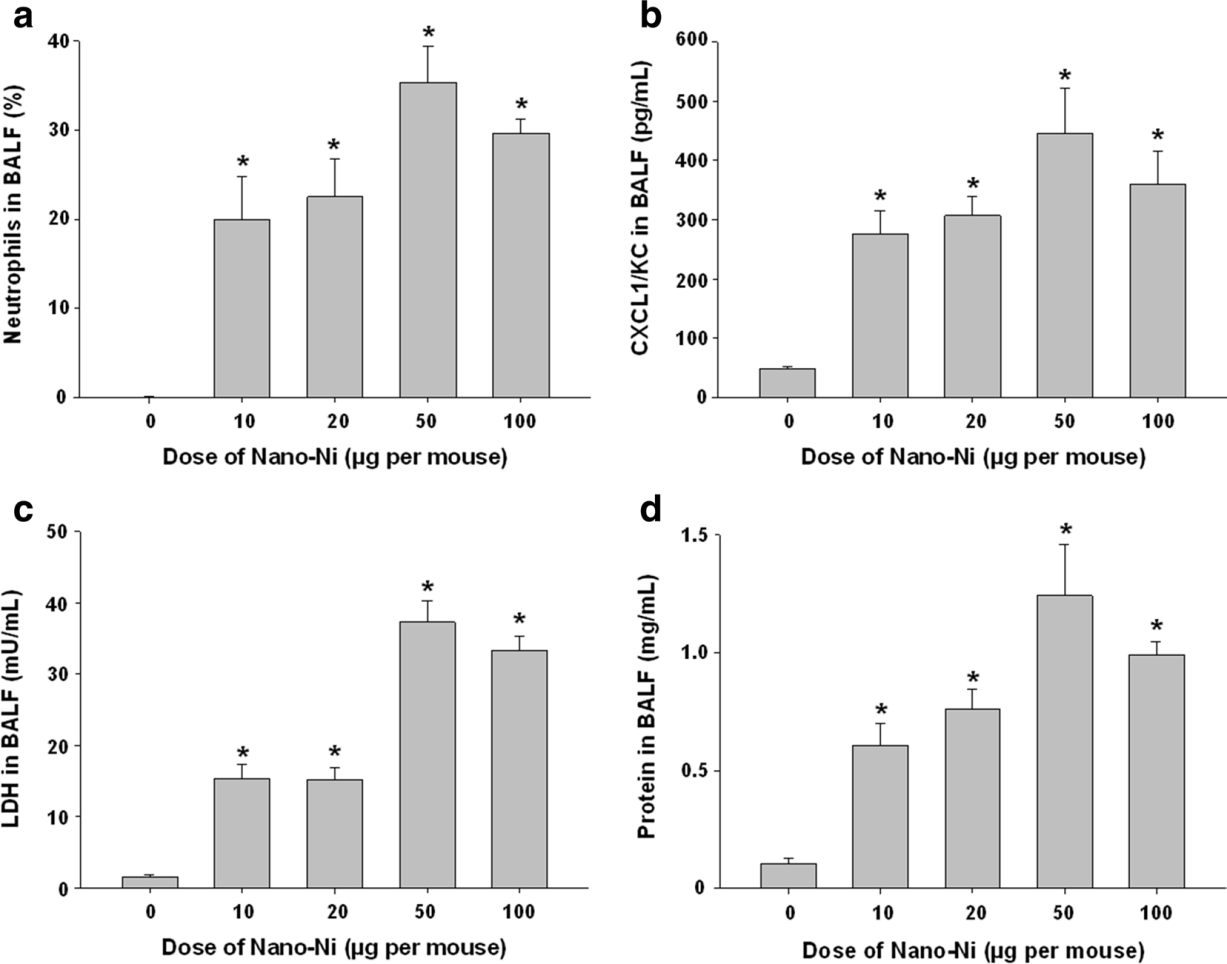
Dose-response study of cellular and biochemical parameters in BALF. Mice were intratracheally instilled with 10, 20, 50, or 100 µg per mouse of Nano-Ni. Control mice were instilled with physiological saline. BALF was collected from mice at day 3 post-exposure. The neutrophil count (**a**), CXCL1/KC level (**b**), LDH activity (**c**), and total protein concentration (**d**) in the BALF were determined. Data are shown as mean ± SE (n = 8 ~ 10). *p < 0.05 vs. Control

In order to find the appropriate dose and time for the comparative study of pulmonary toxicity induced by nickel nanoparticles with differential surface modification, Nano-Ni was used as a “model” nanoparticle to perform dose- and time-response studies. In the dose–response study, mice were exposed to 0, 10, 20, 50, and 100 µg per mouse of Nano-Ni by intratracheal instillation and lavaged at day 3 post-exposure. Figure 2 shows a dose–response increase in the cellular and biochemical constituents in BALF. Even instillation with as low as 10 µg per mouse of Nano-Ni caused acute lung inflammatory response as reflected by increased neutrophil count, total protein concentration, CXCL1/KC level, and LDH activity. 50 µg per mouse of Nano-Ni caused the most severe effects on the cellular and biochemical markers in the BALF. Although there was a slight decrease in these parameters in BALF from mice exposed to 100 µg per mouse of Nano-Ni as compared to those from mice exposed to 50 µg per mouse of Nano-Ni, this decrease was not significant.

**Fig. 3:**
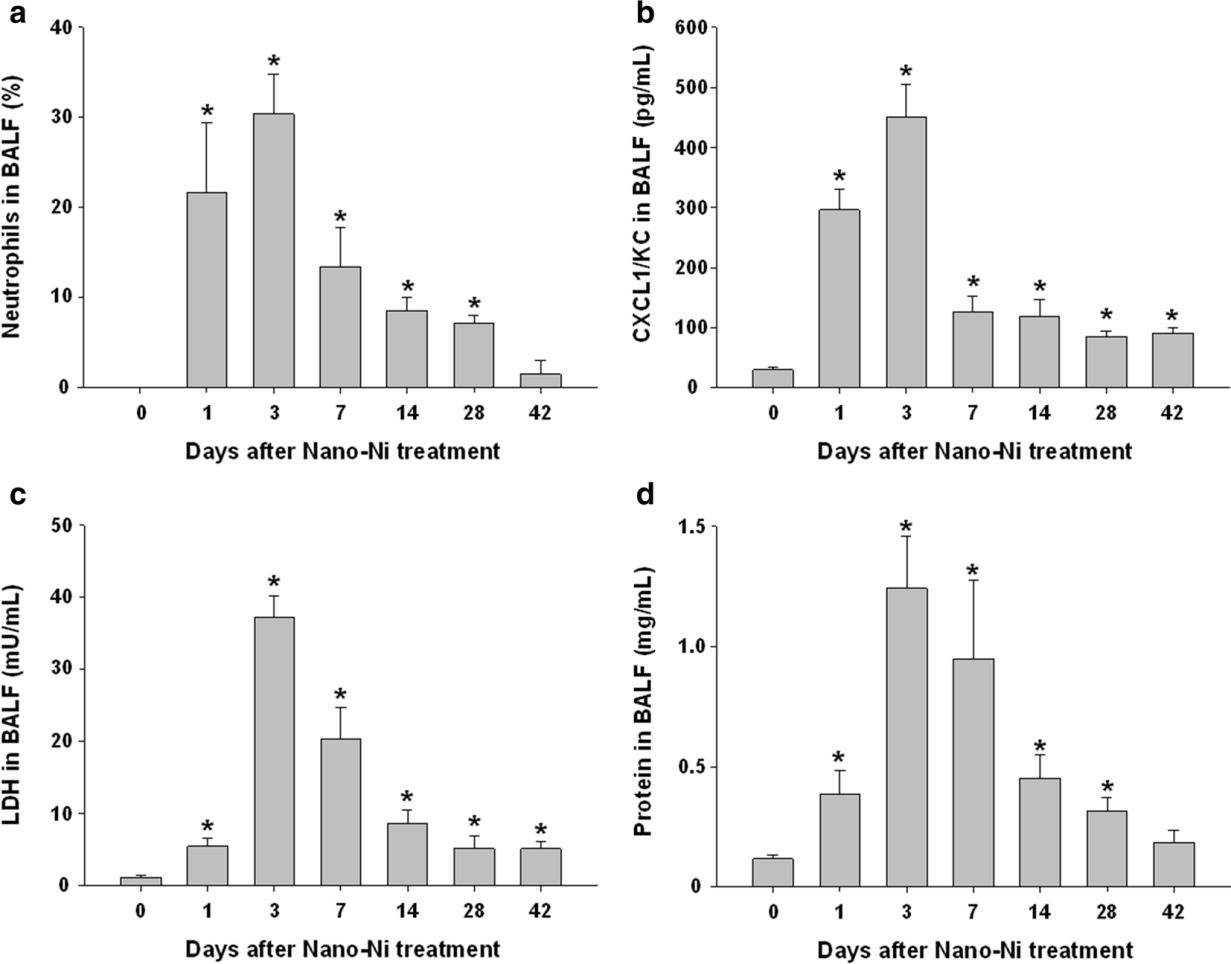
Time-response study of cellular and biochemical parameters in BALF. Mice were intratracheally instilled with 50 µg per mouse of Nano-Ni. Control mice were instilled with physiological saline. BALF was collected from mice at days 1, 3, 7, 14, 28, and 42 post-exposure. The neutrophil count (**a**), CXCL1/KC level (**b**), LDH activity (**c**), and total protein concentration (**d**) in the BALF were determined. Data are shown as mean ± SE (n = 4 ~ 10). *p < 0.05 vs. Control

In the time-response study, mice were exposed to 50 µg per mouse of Nano-Ni, and lavaged at days 1, 3, 7, 14, 28, and 42 post-exposure. No neutrophils or only one or two neutrophils were observed in the lungs of control mice instilled with physiological saline. Nano-Ni instillation caused a marked increase in the number of neutrophils in BALF as early as day 1 post-exposure, which peaked at day 3 post-exposure. The number of neutrophils in BALF decreased from day 3 to day 42; however, the neutrophils in BALF were still observed even at day 42 post-Nano-Ni instillation. The levels of CXCL1/KC, the concentration of total protein, and the LDH activity in BALF had the similar pattern; increased at day 1, peaked at day 3, and decreased from day 3 to day 42 post-instillation, but not reaching the levels of the controls (Fig. 3).

Based on the above dose- and time- response studies, we chose the dose of 50 µg Nano-Ni per mouse for intratracheal instillation, the time point of 3 days post-exposure for the short-term study, and the time point of 42 days post-exposure for the long-term study.

### Acute pulmonary effects with exposure to Nano-Ni, Nano-Ni–P, and Nano-Ni–C

To compare the acute pulmonary effects of nickel nanoparticles with differential surface modification, mice were intratracheally instilled with 50 µg per mouse of Nano-Ni, or with either Nano-Ni–P or Nano-Ni–C with the same molar concentration of Ni as Nano-Ni, and sacrificed at day 3 post-exposure. No mice died during the 3-day experimental period.

#### BALF profiles

**Fig. 4:**
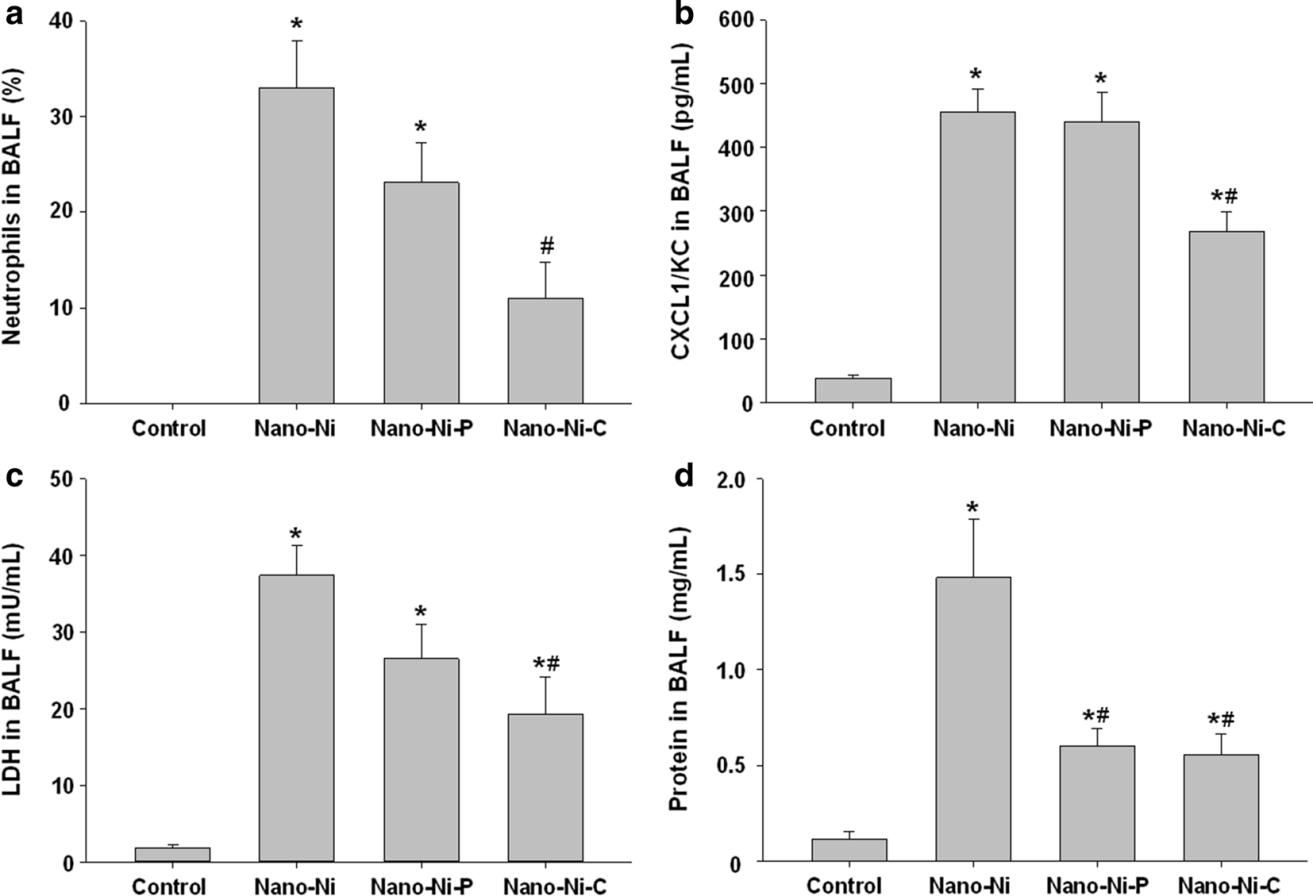
Comparative study of cellular and biochemical parameters in BALF. Mice were intratracheally instilled with 50 µg per mouse of Nano-Ni, or with either partially passivated (Nano-Ni–P) or carbon-coated (Nano-Ni–C) nickel nanoparticles with same molar concentration of Ni as Nano-Ni. Control mice were instilled with physiological saline. BALF was collected from mice at day 3 post-exposure. The neutrophil count (**a**), CXCL1/KC level (**b**), LDH activity (**c**), and total protein concentration (**d**) in the BALF were determined. Data are shown as mean ± SE (n = 5). *p < 0.05 vs. Control; ^#^*p *< 0.05 vs. Nano-Ni group

The cellular and biochemical constituents in BALF were analyzed at day 3 post-exposure of nickel nanoparticles. Our results showed that intratracheal instillation of Nano-Ni or Nano-Ni–P caused severe acute pulmonary inflammatory response and lung injury, which were reflected by a marked increase in neutrophil count, CXCL1/KC level, LDH activity, and the concentration of total protein in BALF obtained from mice 3 days after Nano-Ni or Nano-Ni–P exposure (Fig. 4). Nano-Ni–C exposure also caused pulmonary inflammation; however, to a much lesser extent than that of the mice with Nano-Ni or Nano-Ni–P exposure (Fig. 4). The number of neutrophils, the level of CXCL1/KC, and LDH activity in BALF from mice exposed to Nano-Ni–C were significantly lower than that from mice with Nano-Ni or Nano-Ni–P exposure (Fig. 4).

#### MMP-2 and MMP-9 protein levels and activities in BALF and lung tissues

The gelatinase matrix metalloproteinases (MMPs), MMP-2 and MMP-9, have been implicated in lung inflammation and injury. Increased MMP-2 and MMP-9 in the BALF or tracheal aspirates have been noted in patients with ARDS [43–45]. MMP-2 and/or MMP-9 have also been implicated in the development of experimental acute lung injury [46]. To determine whether the presence of MMP-2 and MMP-9 is associated with lung injury caused by nickel nanoparticle exposure, MMP-2 and MMP-9 protein levels in the BALF were determined by a commercially available ELISA kit, while their activities in both BALF and lung tissue homogenates were analyzed by gelatin zymography assay.

**Fig. 5:**
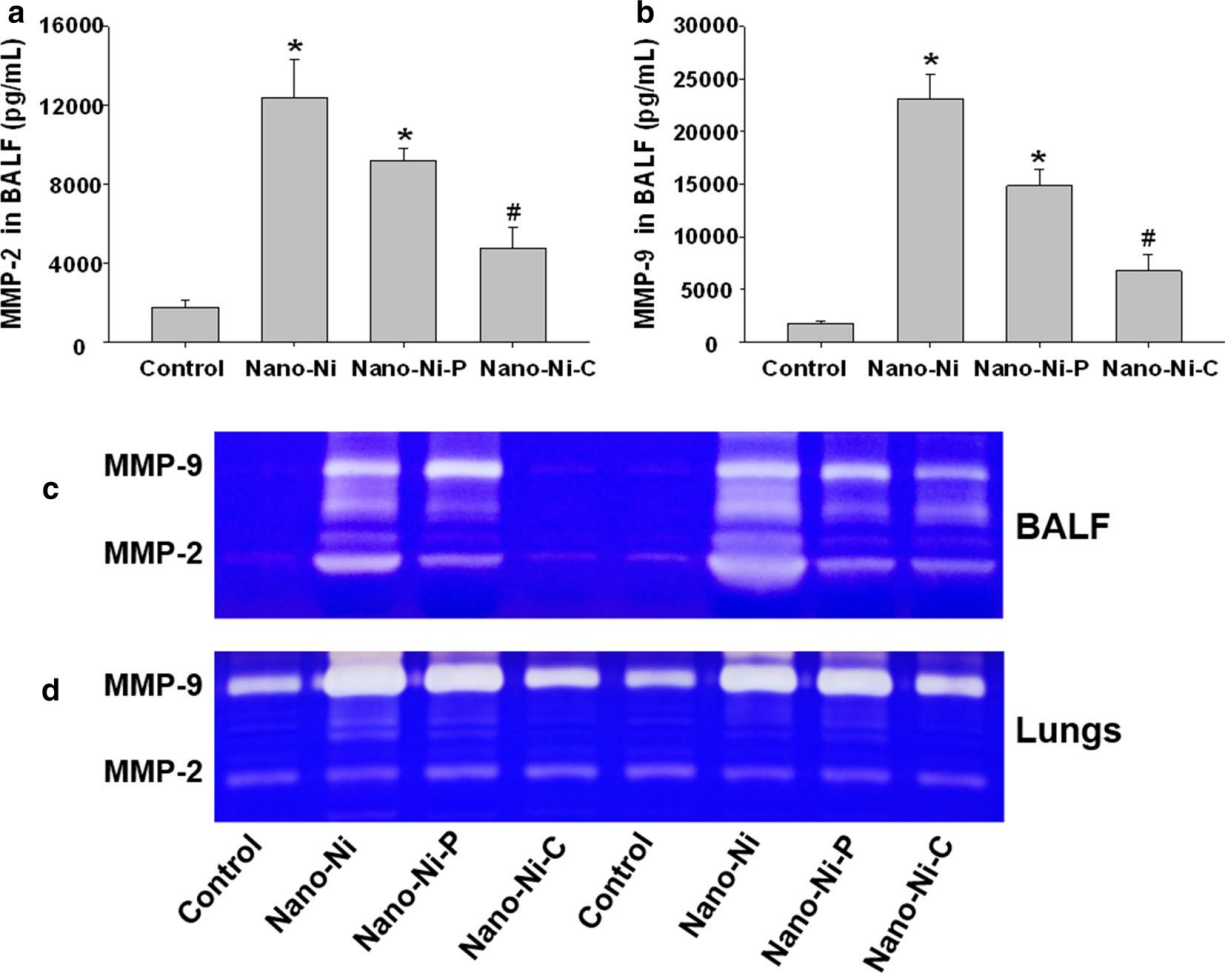
MMP-2 and MMP-9 protein levels (**a**, **b**) and activities (**c**, **d**) in BALF and lung tissues. Mice were intratracheally instilled with 50 µg per mouse of Nano-Ni, or with either partially passivated (Nano-Ni–P) or carbon-coated (Nano-Ni–C) nickel nanoparticles with same molar concentration of Ni as Nano-Ni. Control mice were instilled with physiological saline. BALF and lung tissues were collected from mice at day 3 post-exposure. **a**, **b** MMP-2 and MMP-9 protein levels in the BALF determined by Mouse MMP-2 or MMP-9 PicoKine™ ELISA Kit. Data are shown as mean ± SE (n = 5). *p < 0.05 vs. Control; ^#^*p *< 0.05 vs. Nano-Ni group. **c**, **d** Gelatinolytic activity of MMP-2 and MMP-9 in the BALF (**c**) and lung tissue homogenates (**d**) determined by gelatin zymography assay

Our results showed that exposure to Nano-Ni or Nano-Ni–P led to a significant increase in the MMP-2 and MMP-9 protein levels in the BALF as compared to those of the controls (Fig. 5a, b). However, although Nano-Ni–C exposure caused a slight, but not significant increase in MMP-2 and MMP-9 protein levels as compared with the controls, their levels were significantly lower than that of Nano-Ni- or Nano-Ni–P- exposed mice (Fig. 5a, b). The results of MMP-2 and MMP-9 activities in BALF or lung tissue homogenate analyzed by gelatin zymography assay were consistent with the results of their protein levels in BALF (Fig. 5c, d). Nano-Ni and Nano-Ni–P exposure caused a significant increase in the MMP-2 and MMP-9 activities in both BALF and lung tissues, while Nano-Ni–C exposure only caused a slight, but not significant increase (Fig. 5c, d).

#### Histological examination

**Fig. 6:**
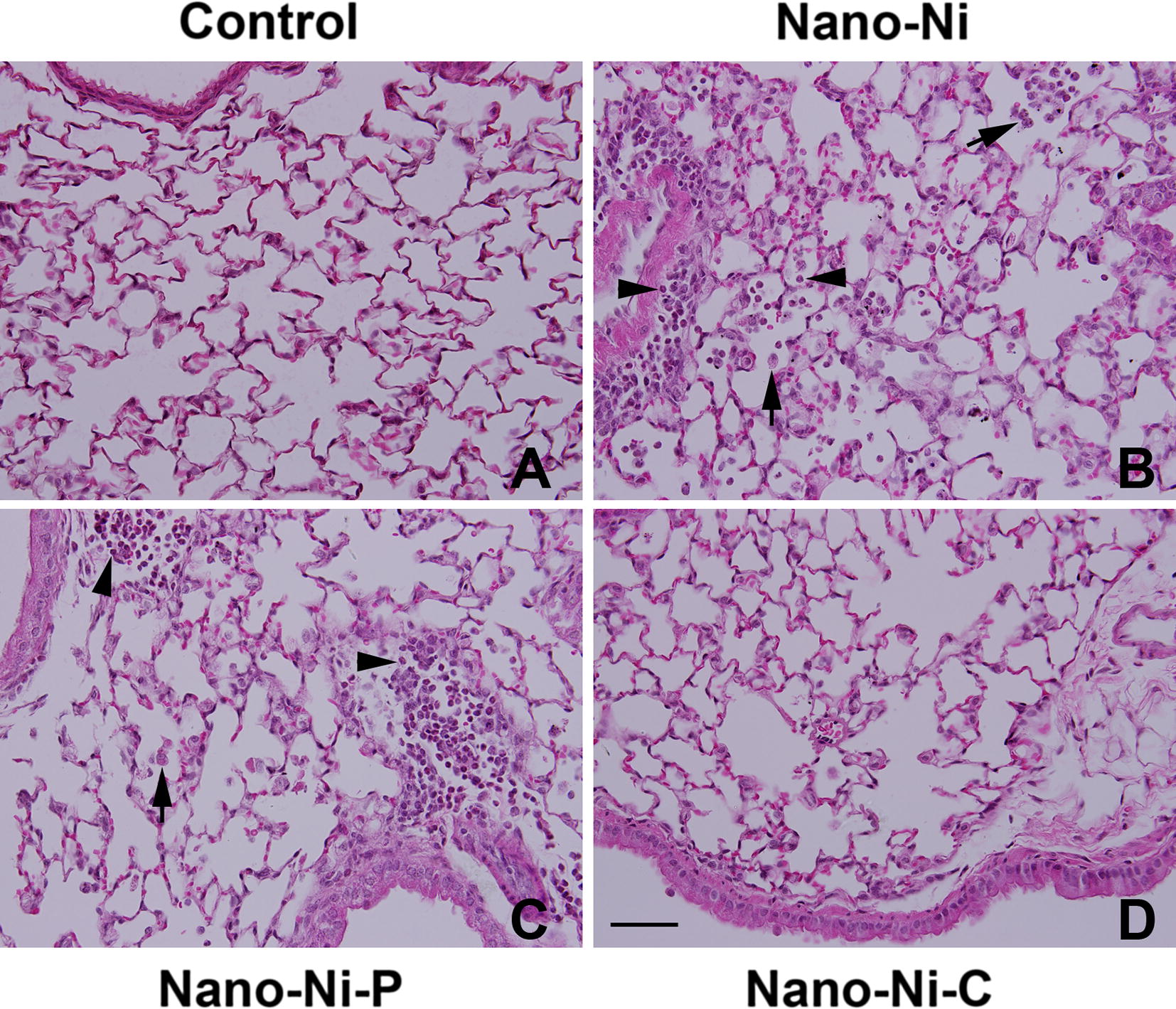
Histology in the lungs of mice 3 days after nickel nanoparticle exposure. Mice were instilled intratracheally with 50 µg per mouse of Nano-Ni, or with either partially passivated (Nano-Ni–P) or carbon-coated (Nano-Ni–C) nickel nanoparticles with same molar concentration of Ni as Nano-Ni. Control mice were instilled with physiological saline. Lung tissue sections collected from mice 3 days post-exposure were analyzed by H&E staining. **A** The normal structure of lung parenchyma in a control mouse.** B** Shows acute inflammation in the lungs of a mouse with Nano-Ni exposure. A large amount of polymorphonuclear (PMN) cells and macrophages infiltration in the perivascular, peribronchial, or peribronchiolar areas, and alveolar space. Acute inflammation was also observed in Nano-Ni–P-exposed lungs (**C**), but very mild in Nano-Ni–C-exposed lungs (**D**). Arrows: macrophages; arrowheads: neutrophils. Scale bar in **D** represents 50 µm for all panels

In order to compare the histopathological changes in the lungs after Nano-Ni, Nano-Ni–P, or Nano-Ni–C exposure, H&E staining was performed on the lung sections collected from mice 3 days post-exposure. In the control mice instilled with physiological saline, normal structure of lung parenchyma was observed. Instillation of Nano-Ni or Nano-Ni–P induced severe acute lung inflammation as evidence by infiltration of a large amount of polymorphonuclear (PMN) cells and macrophages into the mouse lungs; mainly in the perivascular, peribronchial, and peribronchiolar areas, and alveolar space (Fig. 6). Some mice exhibited pulmonary hemorrhage. However, instillation of Nano-Ni–C only induced a mild lung inflammation. Only some neutrophils and macrophages were observed in the mouse lung tissues (Fig. 6).

### Chronic pulmonary effects with exposure to Nano-Ni, Nano-Ni–P, and Nano-Ni–C

To examine whether modification of surface of Nano-Ni could alter Nano-Ni-induced persistent pulmonary inflammation and fibrosis, we compared and examined the lung histopathological changes after exposure to three kinds of nickel nanoparticles. After nickel nanoparticle exposure, the mice in the Nano-Ni-exposed group did not thrive normally. At day 42 post-Nano-Ni exposure, the body weight of mice was still significantly lower than that of the control group (data not shown). However, at day 42 post-Nano-Ni–P and Nano-Ni–C exposure, the body weight of mice were almost similar to that of the control group (data not shown).

#### Histological examination

**Fig. 7:**
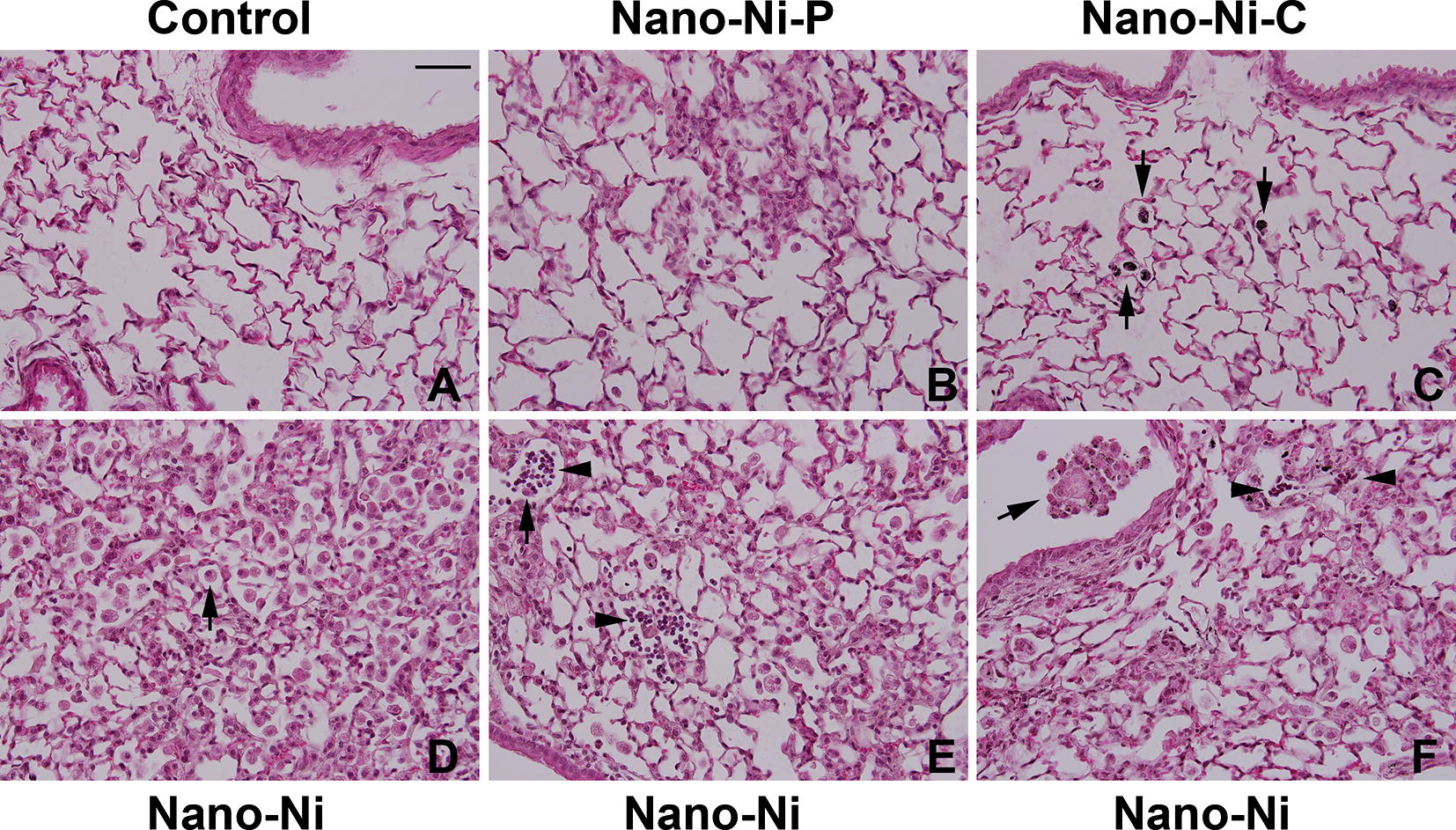
Histology in the lungs of mice 42 days after nickel nanoparticle exposure. Mice were instilled intratracheally with 50 µg per mouse of Nano-Ni, or with either partially passivated (Nano-Ni–P) or carbon-coated (Nano-Ni–C) nickel nanoparticles with same molar concentration of Ni as Nano-Ni. Control mice were instilled with physiological saline. Lung tissue sections collected from mice 42 days post-exposure were analyzed by H&E staining. **A** The normal structure of lung parenchyma in a control mouse. **D**–**F** Extensive chronic inflammation and fibrosis in the lungs of mice with Nano-Ni exposure. A large amount of enlarged and foamy macrophages in the alveolar space (arrow in **D**), alveolar septa, and lumen of bronchi and bronchioles (arrow in **F**), increased number of pulmonary intravascular macrophages (arrow in **E**), thickening of alveolar septa and subepithelial areas of bronchi and bronchioles, and lymphocytes infiltration (arrowheads in **E**, **F**) were observed. Chronic inflammation was also observed in Nano-Ni–P-exposed lungs, but at a much lesser degree (**B**). Only a mild chronic inflammation was observed in Nano-Ni–C-exposed lungs (**C**). Arrows in** C**: particle-phagocytized macrophages. Scale bar in **A** represents 50 µm for all panels

**Fig. 8:**
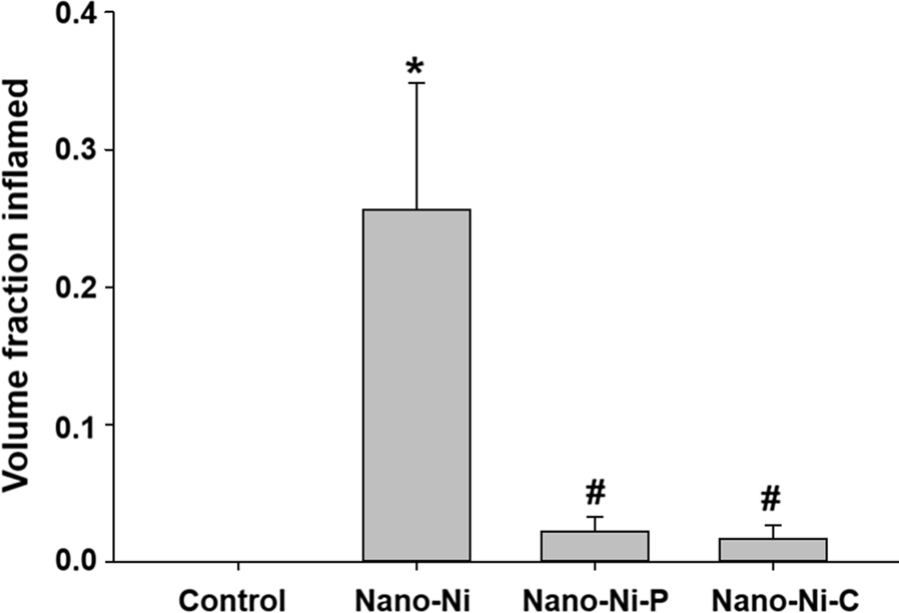
Extent of pneumonitis. Mice were intratracheally instilled with 50 µg per mouse of Nano-Ni, or with either partially passivated (Nano-Ni–P) or carbon-coated (Nano-Ni–C) nickel nanoparticles with same molar concentration of Ni as Nano-Ni. Control mice were instilled with physiological saline. Right lung tissues were collected 42 days post-exposure for histological examination. Data are shown as mean ± SE (n = 5). *p < 0.05 vs. Control; ^#^*p *< 0.05 vs. Nano-Ni group

At day 42 after Nano-Ni, Nano-Ni–P, or Nano-Ni–C exposure, mice were sacrificed and the lung tissues were collected for H&E staining. Normal structure of lung parenchyma was observed in control mice with physiological saline instillation (Fig. 7A). However, instillation with Nano-Ni caused extensive chronic pulmonary inflammation and fibrosis (Fig. 7D–F). A large amount of enlarged and foamy macrophages were observed in the alveolar space (arrow in Fig. 7D), alveolar septa, and lumen of bronchi and bronchioles (arrow in Fig. 7F). Increased number of pulmonary intravascular macrophages was also observed (arrow in Fig. 7E). Nano-Ni exposure caused lung fibrosis, which was reflected by thickening of alveolar septa and subepithelial areas of bronchi and bronchioles (Fig. 7D–F). Lymphocyte infiltration was observed (arrowheads in Fig. 7E, F). Focal chronic inflammation was also observed in Nano-Ni–P-exposed lungs, but at a much lesser degree (Fig. 7B). Only a slight to mild chronic inflammation was observed in Nano-Ni–C-exposed lungs (Fig. 7C), although enlarged, particle-phagocytized macrophages still existed in mouse lungs (arrows in Fig. 7C). The extent of chronic inflammation in the right three lobes was quantified by morphometric analysis, and the results showed that the volume fraction inflamed in the lungs of Nano-Ni-exposed mice was significantly greater than that of the control, the Nano-Ni–P-exposed, or the Nano-Ni–C-exposed mice (Fig. 8).

#### Lung fibrosis

**Fig. 9:**
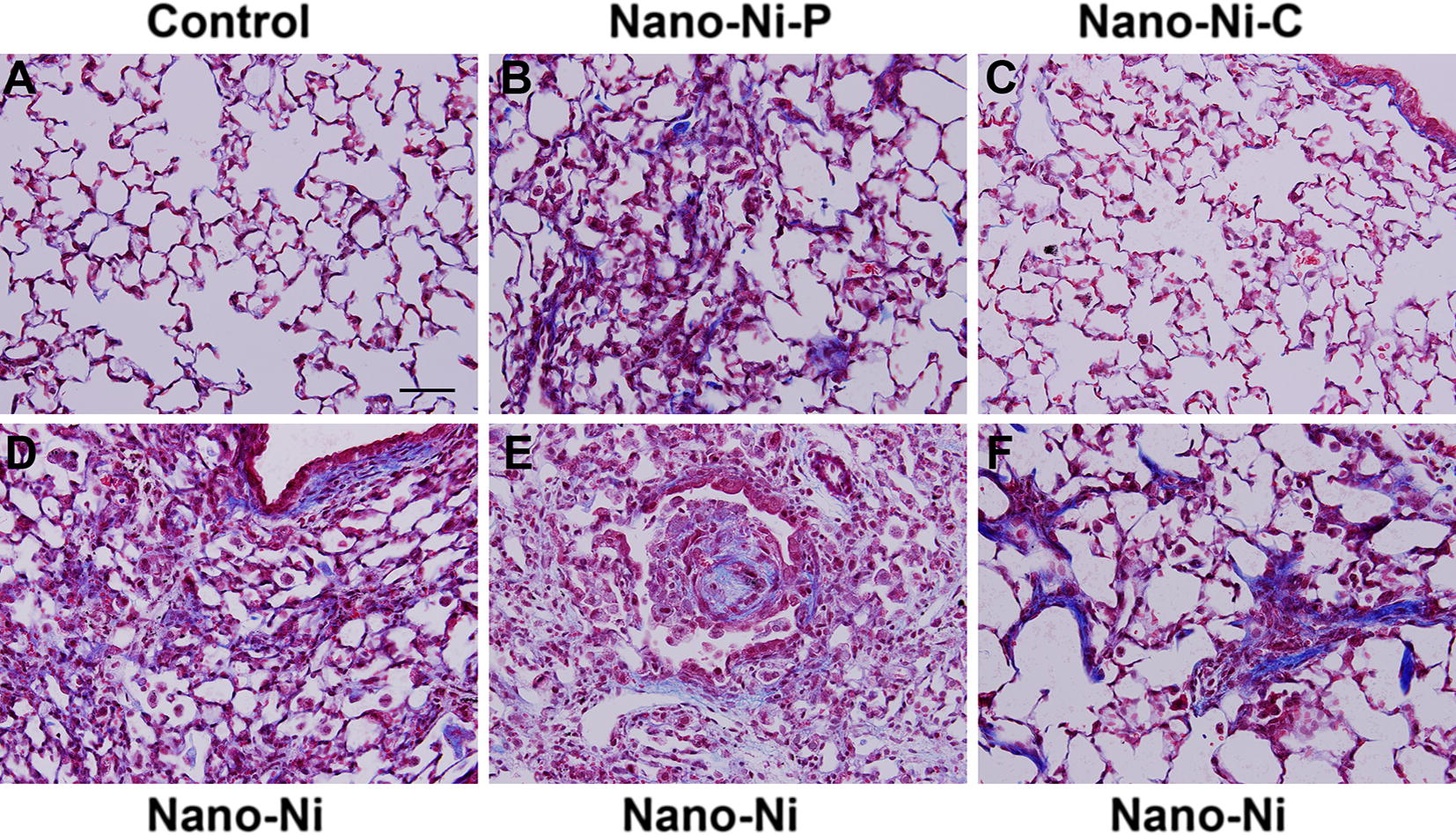
Trichrome staining of the lung tissues from mice 42 days after nickel nanoparticle exposure. Mice were instilled intratracheally with 50 µg per mouse of Nano-Ni, or with either partially passivated (Nano-Ni–P) or carbon-coated (Nano-Ni–C) nickel nanoparticles with same molar concentration of Ni as Nano-Ni. Control mice were instilled with physiological saline. **A** The normal structure of lung parenchyma in a control mouse. **D**–**F** Extensive pulmonary fibrosis (collagen deposition, blue staining) in the mice with Nano-Ni exposure. Fibrosis was also observed in Nano-Ni–P-exposed lungs (**B**), but not in Nano-Ni–C-exposed lungs (**C**). Scale bar in **A** represents 50 µm for all panels

Nano-Ni exposure caused extensive lung interstitial fibrosis at day 42 post-exposure by H&E staining, which was further confirmed by Masson’s trichrome staining. An increase in the collagen deposition in the alveolar septa and the peribronchial, peribronchiolar, and perivascular areas, and proliferation of interstitial cells in the lung tissues were observed in the Nano-Ni-exposed mice (Fig. 9D–F). Fibrosis was also observed in some areas of Nano-Ni–P-exposed lungs, but to a much lesser extent than that of the Nano-Ni-exposed lungs (Fig. 9B). In contrast, no obvious or only a mild fibrotic response was observed in the lungs of Nano-Ni–C-exposed mice (Fig. 9C).

**Fig. 10:**
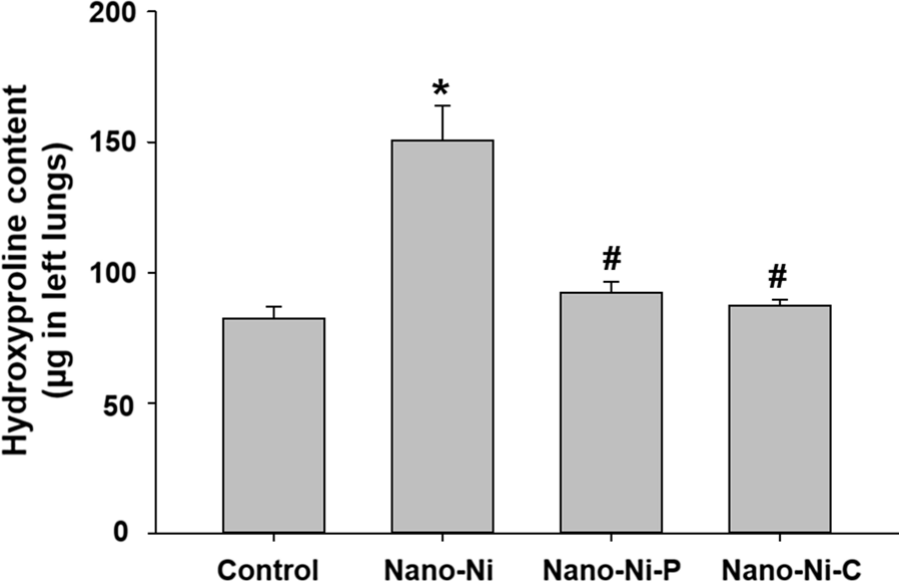
Hydroxyproline content in mouse left lungs. Mice were intratracheally instilled with 50 µg per mouse of Nano-Ni, or with either partially passivated (Nano-Ni–P) or carbon-coated (Nano-Ni–C) nickel nanoparticles with same molar concentration of Ni as Nano-Ni. Control mice were instilled with physiological saline. Left lung tissues were collected 42 days post-exposure for determination of hydroxyproline content. Data are shown as mean ± SE (n = 5). *p < 0.001 vs. Control; ^#^*p *< 0.001 vs. Nano-Ni group

The hydroxyproline content in the mouse left lungs after nickel nanoparticle exposure was also determined. Hydroxyproline is a major component of the protein collagen. Measurement of its content in the lung tissues is the most common way to quantify pulmonary fibrosis [39, 47]. Our results showed that exposure to Nano-Ni resulted in a significant increase in the hydroxyproline content in the left lungs (Fig. 10), which was much higher than that of the Nano-Ni–P- or Nano-Ni–C- exposed lungs (Fig. 10). There was only a slight increase in the hydroxyproline content in the Nano-Ni–P- or Nano-Ni–C- exposed lungs as compared to that of the control lungs, but it was not significant (Fig. 10).

### The effects of nickel nanoparticles on lipid peroxidation and oxidative DNA damage

**Fig. 11:**
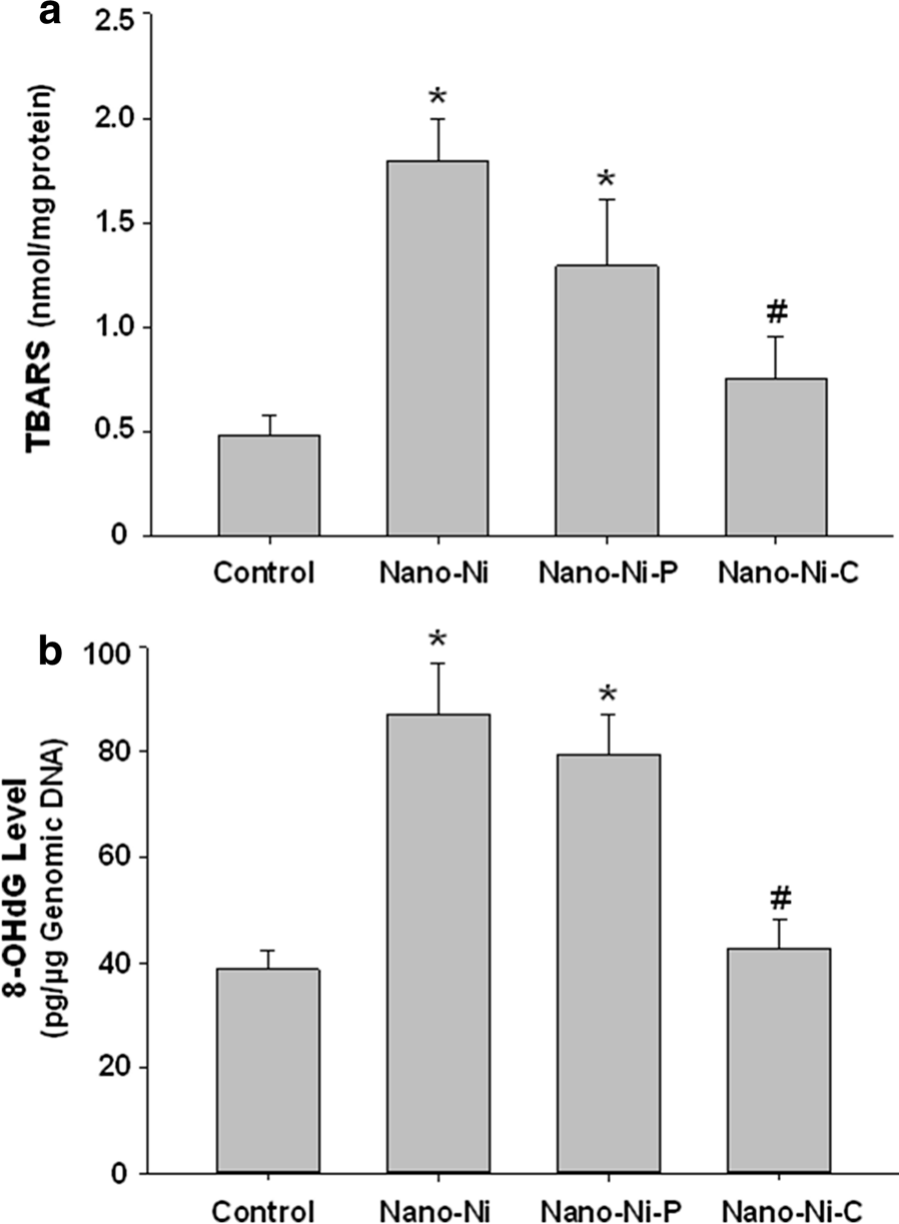
The levels of TBARS and 8-OHdG in mouse lung tissues 3 days after nickel nanoparticle exposure. Mice were instilled intratracheally with 50 µg per mouse of Nano-Ni, or with either partially passivated (Nano-Ni–P) or carbon-coated (Nano-Ni–C) nickel nanoparticles with same molar concentration of Ni as Nano-Ni. Control mice were instilled with physiological saline. Mouse lung tissues were collected 3 days post-exposure. **a** TBARS level in mouse lung tissues. Data represent mean ± SE (n = 5). *p < 0.01 vs. Control; ^#^*p *< 0.05 vs. Nano-Ni group. **b** 8-OHdG level in genomic DNA of mouse lung tissues. Data represent mean ± SE (n = 5). *p < 0.01 vs. Control; # *p *< 0.05 vs. both Nano-Ni and Nano-Ni–P groups

To determine whether exposure to nickel nanoparticles resulted in oxidative damage to lipid and genomic DNA in the lung tissues, the levels of lipid peroxidation and oxidative DNA damage were analyzed in the lung tissues 3 days post-nickel nanoparticle exposure by Thiobarbituric Acid Reactive Substances (TBARS) assay and an OxiSelect™ Oxidative DNA Damage ELISA Kit, respectively. TBARS assay is a well-established assay for screening and monitoring lipid peroxidation, while 8-OHdG is a biomarker of oxidative DNA damage caused by reactive oxygen species (ROS) [48]. Our results showed that Nano-Ni exposure caused significant increases in TBARS and genomic DNA 8-OHdG in mouse lung tissues (Fig. 11). Nano-Ni–P had similar effects as Nano-Ni. However, Nano-Ni–C only caused a slight, but not significant, increase in the levels of TBARS and 8-OHdG as compared to that of the control, and the levels were significantly lower than that of the Nano-Ni-exposed mice (Fig. 11).

## Discussion

Nickel and its compounds play an important role in our daily lives. Compared to other materials, nickel-containing products possess better corrosion resistance, greater toughness, higher strength at high and low temperatures, and other special magnetic and electronic properties [49]. Previous studies have shown that exposure to nickel and nickel compounds cause lung injury and inflammation, pulmonary fibrosis, emphysema, alveolar proteinosis, and lung cancer [15, 50]. The use of Nano-Ni has grown and occupational and non-occupational exposure has increased. Consequently, it is important to understand the potential toxic effects of Nano-Ni and to investigate the factors, such as the physicochemical properties of Nano-Ni, involved in Nano-Ni-induced toxicity. Exploring whether modification of Nano-Ni surface can alter the toxic effects of Nano-Ni will stimulate the scientific community to develop new nickel-containing nanoparticles that have similar properties but less toxicity. Although a previous in vitro study compared the cytotoxic effects of Nano-Ni and carbon-nickel nanoparticles (Nano-Ni/C) [51], few studies focused on their comparative in vivo pulmonary toxicity. While in vitro studies may reveal possible differences in toxicity, in vivo studies provide a broader picture reflecting the overall effects that include the organism’s adaptations to injury. In the present study, we investigated and compared the pulmonary effects of mice exposed to three kinds of nickel nanoparticles with the same diameter and surface area, but with differential surface modification. The goal of this study was to investigate whether surface modification could alter Nano-Ni-induced pulmonary toxicity.

Three kinds of nickel nanoparticles were used in this study and their average particle sizes in the powder are around 20 nm, which were confirmed by TEM. Therefore, these particles are considered as nanoparticles. The size distribution of these nickel nanoparticles in physiological saline was determined by DLS. The results showed that some of the nickel nanoparticles aggregated in the solution. Although several previous studies showed that adding either sodium dodecyl sulfate (SDS) or some protein, such as bovine serum albumin (BSA), can reduce the aggregation of nanoparticles [52, 53], to reduce the aggregation of nanoparticles by these methods is not a process under physiological condition to which a real world individual could potentially be exposed. Because one of the well-documented nanoparticle properties is their ability to aggregate, and human exposures will include exposure to aggregated nanoparticles to some degree, so the use of aggregated nanoparticles in toxicology studies is relevant. It is important to note that even in the aggregated nanoparticles, increased toxic effects are associated with increased nanoparticle surface area [19, 21, 24, 25, 54]. In addition, since the formation of aggregates is through chemical bonds or physical interaction forces, the aggregated nanoparticles can be easily released as a single nanoparticle or smaller aggregated nanoparticles under physiological conditions in the body, such as respiratory movement, stomach and intestine digestion [21, 23, 25, 54]. Therefore, although the hydrodynamic sizes of these nickel nanoparticles in the present study are more than 200 nm, they are still considered as nanoparticles. In fact, in small pieces of lung tissues obtained at autopsy from a patient died of nickel nanoparticle exposure, TEM has demonstrated groups of nickel nanoparticle aggregates in the cytoplasmic lysosomes [17].

Although metal nanoparticles are widely used in industry, exposure limits for many metal nanoparticles, including nickel nanoparticles, are still undefined. It is extremely difficult to predict an accurate occupational exposure to Nano-Ni given the variability of Nano-Ni generation and the ventilation in the worksite. In this study, the dose of 50 µg per mouse of Nano-Ni was selected according to our dose–response study. This dose is equivalent to ~ 0.1 μg/cm^2^ epithelium, assuming a total alveolar surface area of a mouse lung of 500 cm^2^ [55, 56]. The inhaled particles could accumulate over time in the lungs. Accidental exposure to metal nanoparticles in the workplace also cannot be ignored. In a case report involving accidental Nano-Ni exposure [17], a worker was exposed to ~ 1 g of nickel nanoparticles in 90 min, which led to death from acute respiratory distress syndrome (ARDS). Therefore, the doses we used in this study was not unreasonable, although demonstrating toxicity at lower doses of Nano-Ni in our future studies will boost our findings.

BAL and analysis of cellular and biochemical profile of BALF are widely used to evaluate particle-induced alterations in pulmonary epithelial integrity, cell damage, inflammatory cell infiltration, cytokine release, and surface release of cellular secretory products. For example, LDH is a cytoplasmic enzyme that is released extracellularly when cells are damaged. The enzyme activity in BALF may provide a quantitative assessment of cell and tissue damage when lungs are exposed to various particles [57, 58]. Accumulation of neutrophils and macrophages in the lungs are also key events in the inflammatory response to inhaled particles, including metal nanoparticles [2, 3]. In the first part of this study, we performed dose- and time-response experiments with Nano-Ni. Our results showed that exposure to Nano-Ni leads to severe acute lung injury and lung inflammation reflected by increased number of neutrophils, CXCL1/KC, LDH activity, and total proteins in BALF, which was confirmed by histopathological examination. These effects of Nano-Ni were dose-related, and were most severe at 50 and 100 µg of Nano-Ni exposure. Thus, we chose a dose of 50 µg Nano-Ni per mouse to exposure mice for the following comparative studies. Our results also showed that instillation of Nano-Ni induces chronic lung inflammation; the neutrophil count in the BALF was still elevated at day 42 post-exposure. This was accompanied by cell damage and alteration of the alveolar/capillary barrier integrity, as shown by increased LDH activity and total protein concentration in BALF. Exposure to Nano-Ni also caused a significant increase in CXCL1/KC level in BALF. It is well-known that chemokine production is a critical step associated with leukocyte accumulation in the lungs, and CXCL1/KC is considered important neutrophil chemo-attractants released in the lungs in many animal models of airway inflammation, induced by exposure to various particles [34, 59]. Airway accumulation of neutrophils and production of inflammatory mediators, including cytokines and chemokines, are characteristics of an inhaled particle-induced early inflammatory response [34]. Therefore, exposure to Nano-Ni resulting in the increase of CXCL1/KC level in BALF may aggravate the Nano-Ni-induced pulmonary inflammatory response by recruiting and activating neutrophils in the lungs through movement of circulating leukocytes to the lungs. From our results on the kinetics of neutrophils, LDH activity, CXCL1/KC level, and total protein concentration in BALF, it is clear that exposure to Nano-Ni causes high and persistent lung inflammation and injury. In addition, the results from our dose- and time- response studies could provide information of appropriate doses and time points for the subsequent comparative studies.

The toxicity of nanoparticles depends on their physicochemical characteristics, such as the particle size and size distribution, surface area, purity, chemical composition, surface properties, surface reactivity, stability, and surface charge [19, 22, 24, 25, 54]. In the second part of this study, we presented a comparative assessment of pulmonary toxic effects of Nano-Ni, Nano-Ni–P, and Nano-Ni–C, which have the same diameter and surface area, but differential surface modification. Our short-term study results demonstrate that exposure to Nano-Ni, Nano-Ni–P, and Nano-Ni–C causes mild to severe acute lung injury and inflammation, supported by increased neutrophil count, CXCL1/KC level, total protein concentration, and LDH activity in BALF. However, the effects caused by Nano-Ni or Nano-Ni–P were far greater than that of Nano-Ni–C. The results were further confirmed by histopathological examination, which revealed an influx of a large number of neutrophils and macrophages into the pulmonary parenchyma after Nano-Ni or Nano-Ni–P exposure. However, exposure to Nano-Ni–C only induced a few neutrophils and macrophages infiltrating into the lungs.

The matrix metalloproteinases (MMPs), a family of 25 secreted and cell surface-bound neutral proteinases, process a large array of extracellular and cell surface proteins under normal and pathological conditions [60, 61]. The MMP family regulates a multitude of biological responses in the lungs that range from normal development to destructive and fibrotic changes. Among the MMPs, the 72 kDa gelatinase A (or MMP-2) and the 92 kDa gelatinase B (or MMP-9) are believed to be the critical enzymes for degrading type IV collagen, a major component of basement membrane [60–63]. MMPs can act directly by degrading lung ECM and/or other secreted proteins; alternatively, they function indirectly by changing the properties of the cleaved proteins in the alveolar space [62, 63]. MMP-2 and MMP-9 are involved in most inflammatory diseases of the lungs. Potential MMP-2 and MMP-9 targets in the lungs include structural proteins in the extracellular matrix (ECM), cell adhesion molecules, growth factors, cytokines, and chemokines. Therefore, activation of MMP-2 and MMP-9 may play an important role in pulmonary diseases. In fact, our previous in vitro studies showed that exposure of human monocytes U937 to Nano-Co or Nano-Ni caused mRNA up-regulation and increased activities of MMP-2 and MMP-9 [29, 37]. Neutrophils could also release proteases in response to injury, including MMPs [64, 65]. Here, we revealed that exposure of mice to three kinds of nickel nanoparticles caused increases in MMP-2 and MMP-9 protein levels in BALF, and also their activities in both BALF and lung tissues. Nano-Ni or Nano-Ni–P caused significantly higher levels of MMP-2 and MMP-9 than Nano-Ni–C. Matrix-degrading MMP enzymes are not only directly responsible for airway and pulmonary injury and inflammation, but also play an important role in the repair process [60–63]. Previous studies showed a correlation between neutrophil presence and pro-MMP-9 levels in airways after repetitive particulate Cr(VI) exposure [66]. In addition, MMP-9 has been shown to play important roles in other lung diseases and is critical in neutrophilic inflammation after ventilator-induced lung injury [67]. MMP activity may also be altered by environmental pollutants. Rats exposed to a combination of particulate matter and ozone experience increased MMP-2 activity in BALF [68]. Therefore, our results suggest that upregulation of MMP-2 and MMP-9 caused by nickel nanoparticle exposure may be related to Nano-Ni- or Nano-Ni–P-induced severe lung inflammation and injury.

To explore and compare chronic pulmonary effects of three kinds of nickel nanoparticles, in the third part of this study, mice were exposed to Nano-Ni, Nano-Ni–P, or Nano-Ni–C, respectively. At day 42 post-exposure, mouse lung tissues were collected for histopathological examination. Our results showed that exposure to Nano-Ni caused significant persistent chronic lung inflammation that was reflected by the infiltration of a large amount of enlarged and foamy macrophages and neutrophils into the lung parenchyma. Our results also showed that exposure to Nano-Ni caused extensive lung fibrosis. Although chronic inflammation and fibrosis were also observed in Nano-Ni–P-exposed lungs, it was much lesser severe as compared to that of Nano-Ni exposure. Nano-Ni–C exposure only induced mild chronic inflammation, but did not cause fibrotic effects. The Nano-Ni- and Nano-Ni–P- induced lung fibrosis was further confirmed by trichrome staining and measurement of the hydroxyproline content in the left lungs. Several mechanisms are potentially involved in these effects. First, oxidative stress and inflammation are considered important mechanisms for particle-induced lung injury and fibrosis. Exposure to some metal nanoparticles induce oxidative stress in cells, and activation of oxidative stress-responsive transcription factors such as NF-κB and AP-1, together with the depletion of antioxidant defenses, can lead to the release of pro-inflammatory cytokines [48, 69]. Many studies have shown that some nanoparticles have greater free radical activity than other comparable fine particles [3, 21, 22, 70]. The high surface activity, large surface area, and chemical properties of nanoparticles may play an important role in surface-derived ROS generation [21, 22]. Our XRD data showed that Nano-Ni has the highest NiO to Ni ratio among all three-nickel nanoparticles, indicating the instability of Nano-Ni. Nano-Ni–P partially treated under O_2_ condition showed less tendency for further oxidation because the surface was partially pre-passivated by a layer of NiO. Nano-Ni–C has the smallest NiO to Ni ratio indicating the highest stability among all three-nickel nanoparticles. These data suggest that surface modification, such as carbon coating, may change the surface activity of Nano-Ni, which may further change the potential toxic effects of Nano-Ni. Our results also demonstrate that exposure to Nano-Ni causes oxidative stress in lungs which was reflected by increased lipid peroxidation and oxidative DNA damage in the lungs of mice; however, Nano-Ni–C does not.

## Conclusions

Taken together, our study herein demonstrates that Nano-Ni, Nano-Ni–P, and Nano-Ni–C may cause lung injury and inflammation; however, their extent of pulmonary toxicity is different. The results support that surface modification (surface oxidation or carbon coating) by altering their physicochemical properties may cause differences in their pulmonary effects. The Nano-Ni-induced oxidative stress may be alleviated through surface modification, such as oxidation in the surface or coating with carbon, which may further reduce Nano-Ni-induced pulmonary toxicity. The results provide further understanding of the potential pulmonary toxicity of nickel nanoparticle exposure. However, other variables such as differences in manufacturing methods, source, and contaminants cannot be ignored.

## Abbreviations

BAL: bronchoalveolar lavage
BALF: bronchoalveolar lavage fluid
Nano-Ni: nickel nanoparticles
Nano-Ni–P: partially passivated nickel nanoparticles
Nano-Ni–C: carbon-coated nickel nanoparticles
CXCL1/KC: chemokine (C-X-C motif) ligand 1/keratinocyte chemoattractant
LDH: lactate dehydrogenase
MMP-2: matrix metalloproteinase-2
MMP-9: matrix metalloproteinase-9
TBARS: thiobarbituric acid reactive substances
8-OHdG: 8-hydroxy-2′-deoxyguanosine
TEM: transmission electron microscopy
XRD: X-ray diffraction
